# Discourse in aging: Narrative and Persuasive

**DOI:** 10.1590/1980-57642018dn13-040012

**Published:** 2019

**Authors:** Zahra Babaei, Zahra Ghayoumi-Anaraki, Behrooz Mahmoodi-Bakhtiari

**Affiliations:** 1 MSc, Department of Speech Therapy, University of Social Welfare and Rehabilitation Sciences, Tehran, Iran.; 2 PhD,Department of Speech Therapy, School of Paramedical Sciences, Mashhad University of Medical Sciences, Mashhad, Iran.; 3 PhD, Department of Performing Arts, University of Tehran, Tehran, IR Iran.

**Keywords:** aging, discourse, narration, persuasion, envelhecimento, discurso, narração, persuasão

## Abstract

The growth in the elderly population has posed a social, economic and health challenge for the twenty-first century.

**Objective:**

Aging is often characterized by changes in cognitive functions which affect the receptive and expressive capabilities of language. Since language plays a significant role in human life, we evaluate the existence of age-related differences in narrative and persuasive discourses.

**Methods:**

The narrative discourse of 91 adults and persuasive discourse of 92 adults,aged from 19 to 75 years and stratified into four age groups,were examined.

**Results:**

There was a statistically significant difference between coherence in the elderly group and each of the other three age groups for both types of discourse. There was also a significant difference for the cohesion variable between the elderly and the first age group for narrative discourse only.

**Conclusion:**

The results of this study demonstrate that discourse is influenced by aging and type (genre) of discourse task. Therefore, it is essential for clinicians to take into account the linguistic needs of elderly and incorporate these into their clinical programs. Also, this finding can help clinicians to distinguish between discourses of normal aging and other neurologic disorders (for example dementia, right hemisphere damage, aphasia).

## RESUMO

O aumento da população idosa colocou um desafio social, econômico e de saúde para o século XXI.

**Objetivo:**

O envelhecimento é frequentemente caracterizado pela presença de alterações nas funções cognitivas que afetam as capacidades receptivas e expressivas da linguagem. Como a linguagem desempenha um papel significativo na vida humana, avaliamos a existência de diferenças relacionadas à idade nos discursos narrativos e persuasivos.

**Métodos:**

O discurso narrativo de 91 adultos e o discurso persuasivo de 92 adultos com idade entre 19 e 75 anos, divididos em quatro faixas etárias, foram examinados.

**Resultados:**

Existe uma diferença estatisticamente significativa entre a coerência no grupo de envelhecimento e cada um dos outros três grupos nos dois tipos de discursos. Existe também uma diferença significativa para a variável coesão entre idosos e a primeira faixa etária apenas no discurso narrativo.

**Conclusão:**

Os resultados deste estudo demonstram que o discurso é influenciado pelo envelhecimento e tipo da tarefa discursiva. Portanto, é essencial que os médicos levem em consideração as necessidades linguísticas dos idosos e as incorporem em seus programas clínicos. Além disso, esses resultados ajudam os médicos a distinguir entre discursos de envelhecimento normal e outros distúrbios neurológicos (por exemplo, demência, lesões do hemisfério direito, afasia).

The growth in the elderly population has posed a social, economic and health challenge for the twenty-first century. According to the World Health Organization (WHO), the number of over-60s as a proportion of the global population is dramatically increasing.^1^ Older age is usually accompanied by a decline in physiological and physical performance.^2^

Aging is often characterized by changes in cognitive function which affect receptive and expressive language abilities.^3^^,^ 4 Paying attention to language dysfunction is of immense importance as it dramatically affects patient communication with others. Language sample analysis is considered one of the best approaches for language skills assessment. In this method, different aspects of the language are analyzed after collecting linguistic samples.^5^ When collecting a linguistic sample, various types of discourse, including descriptive, narrative, procedural, persuasive, interpretive, and conversational discourse can be used.^6^ These discourse types differ according to the linguistic and cognitive demands placed on the individual.^7^

Narrative discourse is a type of discourse involving the expression of events and activities in a temporal sequence^6^ and mostly elicited by visual stimuli, wherein the essential feature includes temporal and causal relations between events in these images.^8^ Picture description is one of the main methods for eliciting narrative discourse,^9^ leading to a better understanding of language skills.^10^ Persuasion is a complex and essential skill, which continues to develop into early adulthood.^11^ In general, compared to narrative discourse, persuasive discourse is considered more cognitively demanding.^12^ Persuasive discourse attempts to express an opinion and give reasons to support that opinion^6^ This type of discourse may occur in both formal situations (e.g. school debates, school essays) and informal situations (e.g. convincing a friend to see a movie or parents to purchase the latest electronic gadget). The ability to persuade and use arguments effectively is considered a fundamental social interaction skill.^13^

In fact, discourse is a complex and dynamic cognitive system in terms of some important processing dimensions: micro-linguistic, macro-structure and macro-linguistic. The micro-linguistic dimension is responsible for inter-sentential functions, the macro-structure is for across-sentence analyses, while the macro-linguistic dimension is responsible for intra-sentential functioning.^14^^,^ 15

The micro-linguistic dimension organizes phonological (or graphemic) sequence chains and words (lexical processing), and determines the syntax needed for each word to make attractive sentences (syntactic processing). The macro-structural level of discourse or cohesion is defined as the set of the possibilities that exists in a sentence for connecting a given statement with previous statements. On the other hand, sentences are conjoined by various kinds of meaning relations described as cohesive ties.^14^ The macro-linguistic dimension determines the meaning of a word or sentence that has a proper context and connects sentences and statements together by means of cohesion and coherence, so as to formulate the main subject of discourse and integrate cognitive and linguistic characteristics.^15^ In other words, coherence refers to overall stability in discourse. Hence, overall coherence indicates how each sentence in a discourse sample is related to the overall subject of the text.^16^

Studies on age-related differences in adult discourse abilities have yielded different results. Although some studies have been conducted regarding the linguistic changes in old age, they have produced conflicting results. Some scholars believe that elderly discourse varies in terms of microstructure.^17^ However, findings of other researchers show that this aspect of language does not change in old age.^3^^,^ 18 Although the findings of other studies reveal that older adults differ from young adults in terms of some microstructural aspects, including semantic paraphasia, no difference in sentence complexity was found.^3^ This inconsistency in results is less present in the macro-structural dimension. Furthermore, according to one study, the number of correct information units (CIU)^19^ and main events^19^^,^ 20 is significantly higher in young people’s discourse than in that of the elderly. Marini et al. also reported that older people produce discourses with significantly less thematic informativeness than young people.^3^

Difficulty in spoken language may erode the ability and desire of adults to communicate, which can affect evaluation of their language capability by themselves and others. Negative self-assessment may promote avoidance of social interaction, whereas being negatively evaluated by others may lead to speech oversimplification. This process can lead to a downward spiral,underscoring the importance of identifying the change in pattern of language during adulthood and old age.^21^

Also, since disorders such as dementia, right hemisphere damage, aphasia, Alzheimer’s disease and other neurologic disorders compromise discourse,^22^^-^ 25 discourse changes during normal aging need to be reviewed. This could help clinicians screen for these disorders in older adults.

Features and rules that influence communication in different languages vary and the results of discourse studies might be affected by these disparities among languages assessed. As an example, in contrast to English, Persian is a pro-drop language with stronger conjugation, where verbs change according to sentence subject.^26^^,^ 27 Therefore, studying discourse features in the Persian language which might be affected by aging, can be of great help to speech and language therapists to better manage elderly people to achieve more effective communication.

Taking into consideration this assumption, the aim of this study is to evaluate the existence of age-related differences in macro-structural and macro-linguistic levels of narrative and persuasive discourse - the two most useful types of discourse- in Persian individuals aged 19 to 75 years of different age groups. 

## METHODS

### Participants

Of 100 subjects invited to participate in the research, 92 subjects took part in the persuasive discourse test and 91 subjects in the narrative discourse test. Participants were stratified into 4 age groups: 19-24, 25-39, 40-60, and 75-60 years. The *World Health Organization* uses *age 60 and older* when referring to elderly populations.^21^^,^ 28

The inclusion criteria for the study were: (a) *to be monolingual* (Persian); (b) having reading and writing literacy skills; (c) no history of stroke or neurological dysfunction; (d) a healthy auditory system; e) a healthy visual system; (f) no history of psychiatric illness, *progressive neurological disease*, non-verbal cognitive *disease* or dementia and depression; (g), no cognitive impairment; (i) being in the 19-74 age group. Participants’ demographic information is presented in Table 1.

**Table 1:** Demographic characteristics of subjects in different age groups.

Study group	Type of discourse	Male	Female	Education (mean±SD)
**1 (19-24 years)**	Narrative	8	31	14.56±1.23
	Persuasive	8	24	13.47±1.52
**2 (25-39 years)**	Narrative	7	14	15.81±1.69
	Persuasive	13	15	15.75±1.79
**3 (40-59 years)**	Narrative	6	15	13.90±3.36
	Persuasive	10	10	13.85±3.58
**4 (60-75 years)**	Narrative	7	3	10.71±3.86
	Persuasive	9	3	10.42±4.56

### Procedures

This is a cross-sectional study and the research protocol was approved by the Ethics Committee of the University of Social Welfare and Rehabilitation sciences. All participants met personally with the examiner to carry out the narrative tasks. The examiner did not impose any time limit, and recorded all sessions for later analysis. Initially, each subject filled out a written consent form regarding participation in the research and personal information.

The overall cognitive functioning of all participants was evaluated by administering the MMSE to ensure that the presence of residual deficits in cognition did not prevent meaningful participation, including the ability to execute the task and comprehend and follow instructions. The MMSE is a brief 30-item questionnaire used for screening cognitive impairments. The maximum score is 30. Cognitive impairment is diagnosed for scores <23.^29^ All participants scored ≥23 in the adopted version of the Mini-Mental State Examination (MMSE) in Persian, thus ensuring they were oriented to self, environment, and place and could participate in the study. A hearing screener confirmed that the included participants had hearing acuity within normal limits. A review of medical records and rehabilitation reports established that visual acuity and perception were sufficient to allow discrimination of pictures. None of the participants had a previous history of psychiatric or neurological illnesses.

Finally, in order to assess the abilities of narrative discourse, the *Persian* Narrative *Discourse Test* was used.^27^ The assessment of narrative skills is performed using story retelling, single picture description or serial picture descriptions. This test uses six serial picture descriptions-all on the same page- to evaluation narrative discourse. The topic of the narrative is a family party. The discourse parameters encoded in narratives will be described later.

Each participant was asked to narrate a story using six serial pictures. In order to avoid poor performance as a result of short-term memory constraints, the pictures were available to participants for viewing until the end of the narration. In narrative assessments using serial or single pictures, these pictures usually remain in front of narrator.^20^ The participants were asked to narrate what they saw in the pictures as a story. A narrative was considered complete when a participant indicated that he/she had nothing more to add. 

The assessment of persuasive abilities was performed using a question. Participants were asked to give an opinion about whether it is better to use public transportation or a private vehicle and why participants would agree or disagree to the claims and reasons presented.^29^ The topic was chosen because it seems to have a widespread appeal and increases the likelihood that participants have personal experiences and ideas. For feedback, the examiner responded only with encouragement to further the discourse (like “Anything else?”) and natural conversational acknowledgements. A discourse was complete when the subject expressed that he/she had nothing more to add.

While expressing the narrative and persuasive discourses, the speech of participants was recorded and then statements were transcribed. These transcriptions included phonemes, pauses, false primers,outsiders and phrases.

Finally, transcribed discourses were analyzed for the cohesion and general coherence indicators.

### Linguistic measures

Connection between utterances in discourse is made by grammatical, conceptual or lexical ties called cohesion. These cohesive ties, as stated by Halliday and Hasan, are categorized into five classes: substitutions, ellipses, references, conjunctions, and lexical markers.^30^ Cohesion is also considered the macro-structural level of discourse.^14^ Analysis of cohesion in this study involves a measure of cohesion per C-unit, in which the cohesion is the total number of all cohesive ties counted in each discourse sample. A C-unit is ‘an independent clause with its modifiers’. It includes one main clause with all subordinate clauses attached to it.^31^

Another measure is global coherence or macro-linguistic level of discourse, which refers to the correlation of the meaning or content of an utterance to the general topic of the story.^31^^,^ 32 In other words, discourse coherence reflects the listener’s ability to interpret the overall meaning conveyed by the discourse. In this study, the four-point scale devised by Wright et al.^32^ (Table 2) is used to score the ability of participants to establish global coherence. An individual score is given to each C-unit, and then the average global coherence score is calculated.

**Table 2:** Scoring criteria for 4-point global coherence rating scale.32

Score	Criteria
1	The C-unit is overtly related to the stimulus as defined by the mention of objects, actors, and actions present in the pictures, which are of significant importance to the main details of the stimulus
2	The C-unit is related to the stimulus, but with some inclusion of suppositional or tangential information that is relevant to the main details of the stimulus; or substantive information is not provided, so that the topic has to be inferred from the statement
3	The C-unit is only remotely related to the stimulus, with probable inclusion of improper egocentric information; it may include tangential information or reference some component of the stimulus that is regarded as non-critical
4	The C-unit is completely unrelated to the stimulus; it may be a comment on the discourse or tangential information

### Reliability

Inter-rater reliability for encodings was determined for the 10%of randomly selected recorded samples. A trained independent speech therapist transcribed and rescored 10% of there corded discourses for assessing inter-rater reliability. 

### Statistical analysis

The Kolmogorov-Smirnov test was used to check the normality of the data. After confirmation of the normality of the data by the Kolmogorov-Smirnov test and the homogeneity of the variance of discourse cohesion, ANOVA and the *Scheffe post-hoc* test were used. Also, Kruskal-Wallis and Mann-Whitney tests were used for coherence, which had a non-normal distribution. Inter-rater reliability was analyzed by Cohen’s Kappa test. Data analysis was performed using SPSS software version 16 and the significance level was set at 0.05.

## RESULTS

In this study, a narrative discourse of 91 adults and persuasive discourse of 92 adults aged between 19 and 75 year were examined. The mean and standard deviation of each of the variables have been presented in Table 3. The results of ANOVA test show that cohesion in narrative discourse differed statistically among groups (P<0.01) (Table 4).Also, the results of the *Scheffe post-hoc* test indicated that this variable differed between the elderly group and first age group only. 

**Table 3:** Mean and standard deviation of values measured for subjects in different age groups.

Variable	Age group 1 (19-24 years)	Age group 2 (25-39 years)	Age group 3 (40-59 years)	Age group 4 (60-75 years)
Cohesion (narrative)	N=39	N=21	N=21	**N=10**
	1.407 ± 0.30	1.211 ± 0.34	1.306 ± 0.47	**0.901 ± 0.45**
Coherence (narrative)	5.00 ± 0.00	5.00 ± 0.00	4.69 ± 0.60	**3.61 ± 1.45**
Cohesion (persuasive)	N=32	N=28	N=20	**N=12**
	1.58 ± 0.62	1.52 ± 0.67	1.23 ± 0.66	**1.23 ± 0.46**
Coherence (persuasive)	5.00 ± 0.00	5.00 ± 0.00	4.69 ± 0.60	**4.42 ± 0.46**

**Table 4:** Significance levels for macrostructure level (cohesion) in different age groups.

	Sum of squares	Mean squares	df	F Value	P Value
Narrative discourse	2.03	0.67	3	4.84	0.004
Persuasive discourse	2.19	0.73	3	1.82	0.14

Regarding coherence, the results of the Kruskal-Wallis test and its post-hoc test showed that coherence differed significantly between the elderly group and all other age groups on both narrative and persuasive discourses (P<0.01) (Table 5), but there were no significant differences between the other 3 groups.

**Table 5:** Significance levels for macrolinguistic level (coherence) in different age groups.

	χ^2^	df	P Value
Narrative discourse	36.24	3	0.0001
Persuasive discourse	14.68	3	0.002

### Reliability

The correlation coefficient for inter-rater reliability was >80% for all variables measured (p<0.05).

## DISCUSSION

The purpose of the current study was to compare the narrative and persuasive discourses of four groups of healthy adults in order to test age-related changes in macro-structural (cohesion) and macro-linguistic level (coherence) measures using a narrative discourse and persuasive discourse task. The analysis of data indicated that aging had an impact in reducing the macro-linguistic level for both types of discourse examined, but there was a selective effect on macro-structural level according to type of discourse.

The measure of cohesion reflects how a participant connects his/her utterances and ideas, by means of different kinds of cohesive ties in the surface structure of discourse for a listener to follow. In this study, older participants had narrative discourses with significantly fewer cohesive ties per C-unit compared to the other age groups. This result may suggest that the older group had difficulties (conceptually and linguistically)linking their utterances in narrative discourse, but not in persuasive discourse. The type of discourse task has been cited among the factors that influence an individual’s performance.^7^ This result is likely due to the fact that, unlike persuasive discourse tasks, narrative discourse uses picture sequences to facilitate the organization of the story structure by visually providing the temporal and logical sequence of events, making the establishment of cohesion easier in stories from picture sequences.^3^^,^ 9 However, older adults cannot exploit these structural aids for the formation of discourse as much as other participant groups.

In the study by Glosser and Desser, no difference was found in the use of cohesive ties in the narrative discourses.^33^ It is noteworthy that, in their study, the narrations were elicited via an informal interview, but in the current study,sequential pictures elicited the narrative discourse.

Coherence is defined as relationships between the meanings underlying the surface structure of a discourse. It refers to a cognitive representation that reflects the interaction between linguistic/discourse characteristics and world knowledge.^33^^,^ 34 On the other hand, impairments in a number of cognitive tasks (working and long-term memory) are seen as part of the normal ageing process in humans.^34^^,^ 35 It has been proposed that executive functions may be an important contributor to discourse generation.^35^^,^ 36 Accordingly, this supra-sentential level of discourse is expected to be affected by aging. 

The findings of the present study of decreased discourse ability for average cohesion and coherence during aging are in line with previous studies^3^^,^ 19 ^,^ 33 ^,^ 37 ^-^ 39 indicating decline in discourse performance with age.

Although Mackenzie showed that educational level does not affect the narrative discourse of individuals with primary and higher education.^40^ Because of the heterogeneity in educational level of the different age groups in the study, the results should be interpreted with caution. Additionally, as the sample size of the elderly group was small, the generalization of findings should be made with more caution.

To conclude, in general, the results of this study demonstrated that discourse is influenced by aging and type (genre) of discourse task. The elderly group had more difficulty on the narrative discourse. Therefore, it is essential for speech-language pathologists to take the linguistic and cognitive needs of elderly individuals into account and incorporate these into their clinical programs.
